# A controlled ‘before-after’ study: impact of a clinical guidelines programme and regional cancer network organization on medical practice

**DOI:** 10.1038/sj.bjc.6600057

**Published:** 2002-02-01

**Authors:** I Ray-Coquard, T Philip, G de Laroche, X Froger, J-P Suchaud, A Voloch, H Mathieu-Daudé, B Fervers, F Farsi, G P Browman, F Chauvin

**Affiliations:** Centre Léon Bérard, 28, rue Laënnec 69008 Lyon, France; Clinique La Digonnière, 60, rue Robespierre 42000 Saint-Etienne, France; Centre hospitalier de Chambéry, BP 1125 73011 Chambéry, France; Centre hospitalier de Roanne, 28, rue Charlieu 42300 Roanne, France; Clinique de Rillieux, 941 rue Capitaine Julien 69140 Rillieux, France; Centre Val d'Aurelle 34094 Montpellier cedex, France; Fédération Nationale des Centres de Lutte Contre le Cancer. 101, rue Tolbiac, 75654 Paris, France; UMR-CNRS (n°5 823), 28, rue Laënnec 69008 Lyon, France; Department of Clinical Epidemiology and Biostatistics, McMaster University, Hamilton, Ontario, Canada

**Keywords:** cancer network, medical audit, medical practice, evidence-based medicine

## Abstract

A regional cancer network has been set up in the Rhône-Alpes region in France. The aim of the project is to improve the quality of care and to rationalize prescriptions in the network. In this network, we assessed the impact of the implementation of a clinical practice guidelines project by assessing the conformity of practice with the guidelines and comparing this with the conformity in an external matched control group from another French region without a regional cancer network. Four hospitals (private and public) accepted to assess the impact of the clinical practice guidelines on the management of breast and colon cancer in the experimental group and three hospitals (private and public) in the control group. In 1994 and 1996, women with non-metastatic breast cancer (282 and 346 patients in the experimental group, 194 and 172 patients in the control group, respectively) and all new patients with colon cancer (95 and 94 patients in the experimental group, and 89 and 118 patients in the control group, respectively) were selected. A controlled ‘before-after’ study, using institutional medical records of patients with breast and colon cancer. The medical decisions concerning the patients were analyzed to assess their compliance with the clinical practice guidelines. When medical decisions were judged to be non-compliant, we verified if they were based on scientific evidence in a published article, if they were not, the medical decision was classified as having ‘no convincing supporting scientific evidence’ The compliance rates were significantly higher in 1996 than in 1994 in the experimental group; 36% (126 out of 346) *vs* 12% (34 out of 282) and 46% (56 out of 123) *vs* 14% (14 out of 103) (*P*<0.001) for breast and colon cancer, respectively. Whereas, in the control group the compliance rates were the same for the two periods; 7% (12 out of 173) *vs* 6% (12 out of 194) (*P*=0.46) and 39% (49 out of 126) *vs* 32% (31 out of 96), *P*=0.19. In the experimental group, in 1994, 101 of the 282 medical decisions (36%) and 27 of the 103 (26%) for breast and colon cancer, respectively, were classified as having ‘no convincing scientific evidence’ compare with 72 out of 346 in 1996 (21%) for breast cancer, and 21 of the 123 (17%) for colon cancer *P*<0.05. Whereas in the control group these results were 106 out of 194 in 1994 (55%) and 90 out of 172 in 1996 (52%), *P*=0.65 for breast cancer and 28 out of 96 in 1994 (29%) and 30 out of 126 in 1996 (24%), *P*=0.36 for colon cancer. The development and implementation strategy of the clinical practice guidelines programme for cancer management results in significant changes in medical practice in our cancer network. These results would suggest that introducing guidelines with specific implementation strategy might also increase the compliance rate with the guideline and ‘evidence-based medicine’.

*British Journal of Cancer* (2002) **86**, 313–321. DOI: 10.1038/sj/bjc/6600057 www.bjcancer.com

© 2002 The Cancer Research Campaign

## 

It has been shown that the simple distribution of guidelines and published reports from consensus conferences does not change physicians' practice (Davis *et al*, 1992; Ferguson, 1995). Input to the development of guidelines from local physicians, dissemination of new information by local opinion leaders, guidelines or algorithms linked with patients' records, patients' reminders, outreach visits and chart review with feedback have all been inconsistently successful in enhancing physicians compliance with guidelines (McDonald *et al*, 1984). The successful introduction of clinical guidelines leading to significant improvements in clinical care depends on many factors including the clinical setting (Grilli and Lomas, 1994), and the methods of development, dissemination (Eddy, 1991), and implementation of the guidelines (Eddy, 1990). Clinical Practice Guidelines (CPGs) is a project initiated by the Centre Léon Bérard, a comprehensive cancer centre in Lyon (France), as part of the institution's quality management programme. The development, implementation and evaluation of the impact of the CPGs on medical practice in one comprehensive cancer centre have already been reported (Ray-Coquard *et al*, 1997). An increased number of medical decisions compliant with the CPGs were reported in this study (Ray-Coquard *et al*, 1997). At the same time as this previous study, the cancer centre provided leadership in the Rhône-Alpes region for cancer treatment and developed a managed care network model similar to those previously described (Siren and Laffel, 1996). This approach was initiated in 1995, when hospitals located in the Rhône-Alpes region in France were invited to participate in the elaboration of a regional cancer network: ONCORA (ONCOlogy Rhône-Alpes). The aims of this regional project, which was organized on the basis of voluntary participation, were: (1) to assist hospital-based oncologists in their decision-making (Fink *et al*, 1984); (2) to minimize inappropriate variation in practice (Farrow *et al*, 1992); (3) to optimize health benefit; and (4) to rationalize prescriptions of chemotherapy. The cancer network development plan involved the implementation of the CPGs elaborated in the cancer centre.

We conducted a controlled ‘before-after’ study to evaluate the impact of the CPGs and the implementation strategy in the cancer network. An external medical record audit process was set up in the two regions to assess the impact of CPGs on the diagnostic and treatment practice and for breast and colon cancer: Rhône-Alpes, with its regional cancer network organization, was the experimental group, and an anonymous matched control region which does not have either a regional cancer network, a process of guidelines implementation, or structures for co-ordinating clinical practice at the time of the data collection, was the control group.

## METHODS

### Objectives

We assessed the impact of the introduction of the CPGs in the regional cancer network on the management of breast and colon cancer by comparing the compliance of medical practice with the CPGs between the two matched groups of hospitals. When medical decisions were judged to be non-compliant, we verified if they were based on scientific evidence in a published article.

### Criteria for hospitals, region and tumours selection

#### Regions

The experimental region is Rhone-Alpes (France), where the CPG programme was implemented, which belongs to Frances's regional cancer network: ONCORA. The control region was one that did not participate in the CPG program, or in the ONCORA regional cancer network organization. The control region was selected to be similar to the experimental region with respect to the presence of medical school training and teaching hospitals (although teaching hospitals were excluded), a mix of private and public institutions, and more than 5000 newly diagnosed cancers reported annually.

#### Institutions

Each group had to include institutions that were from both private and public sectors. To be eligible, each hospital had to be able to produce at least 50 new patient records treated for breast or colon cancer for each time period, and participation was voluntary. To qualify for the experimental group, a hospital in the Rhone-Alpes region had to be participating in regional cancer network activities since 1995.

#### Tumors

Localized breast cancer and colon cancers (of any stage) were selected because of the adapted representation of these two tumours for the medical practice in oncology and in the network (frequency, numerous sources of evidence for breast cancer, few for colon cancer).

### Setting

#### Experimental group

ONCORA is a cancer network located in the Rhône-Alpes region (France). This region (with an area of 43 500 km^2^ and 5.5 million inhabitants) has about 100 public hospitals (with four teaching hospitals) and 87 private hospitals, providing a total of about 26 000 beds). The ONCORA network covers 11 private and 15 public hospitals within this region with a total of about 5 500 beds. Approximately 17 000 new malignant tumours are treated annually in the Rhône-Alpes area and 40% are treated within the organized cancer network. Because of the participating date of 1995, and the number of required records for breast and colon cancer, only four hospitals satisfy the selection criteria (three public and private structures) and all of them accepted to participate in the evaluation of the impact of the CPGs. These four structures were financially organization, and geographically typical of ONCORA as a whole.

#### Control group

The control region (with an area of 27 000 km^2^ and 2.3 million inhabitants) has about 40 public hospitals (with three teaching hospitals) and 80 private hospitals, providing a total of about 11 000 beds). Approximately 9000 new malignant tumours are treated annually in this region. Three of the four hospitals with an activity sufficient to satisfy the selection criteria, agreed to participate (two public and one private structures).

### Intervention: cancer network development and implementation strategy of the CPGs

Physicians and administrative staff from 26 public or private general hospitals of the 40 hospitals with a sufficient level of oncology activity located in the experimental region, agreed to participate in the network and accepted the principles of the implementation of CPGs and evaluation by an external audit procedure. The implementation of the CPGs involved monthly meetings in 1995. Each meeting concentrated on a specific cancer site. Local opinion leaders (from the cancer centre), who were both knowledgeable and credible for the specific cancer site, presented the relevant sections of the CPGs. The information was then discussed, modified and/or validated by all the participating physicians from the 26 hospitals to obtain a regional consensus. Two weeks after the meeting, the validated CPGs were sent to all the participating physicians who were expected to use them in their practice. However, no specific penalty or reward system was included in this implementation strategy. At the end of 1995, all the participating physicians agreed to publish the CPGs as a booklet and a computer programme, which can be integrated in hospital information systems (Centre Léon Bérard Réseau ONCORA, 1997).

### Study design

This controlled ‘before-after’ study, using institutional records of breast and colon cancer patients was conducted in 1994 (the period ‘before’ implementation of the CPGs in ONCORA), and in 1996 (the period ‘after’ when the CPGs had been fully implemented for 1 year). These 2 years were selected because the ‘state-of-the-art’ treatment had not been dramatically modified over this period. This stability is witnessed by the lack of publications of substantial results of studies concerning the management of localized breast cancer over this period. However, for colon cancer, the results of a highly positive randomized controlled trial in metastatic disease were presented in 1995 (de Gramont *et al*, 1995) and published in 1997 (de Gramont *et al*, 1997), and this may have resulted in a modification of medical practice. The analyses for colon cancer were, therefore, stratified according to a modified Duke's classification to control for this potential bias.

### Data sources

The data were collected (by one of the authors (I Ray-Coquard, medical oncologist and a technician) and analyzed by one of the authors (I Ray-Coquard) working independently of the practitioners caring for patients in the different hospitals. It was impossible to do this analysis blinded to the year of treatment since the data were obtained directly from the patients' records. To control the extracted data, a random 10% sample was rechecked by the same ratter, blinded to the previous decision. The physician agreement rate with the original decision was 95% (kappa test=0.89). In addition, the experts from the Centre Léon Bérard confirmed the decisions for this sample.

### Selection of patients' records

For this kind of study, at least 50 patient's records were necessary to detect a statistically significant increase in the compliance rate. Because of the recruitment of the institutions, all the records of localized breast cancer were selected in five of the hospitals, but in two (one in the control group, one in the experimental group), due to their high recruitment, a computerized procedure (random numbers) was used to select 100 from about 300 women with localized breast cancer for both years. Randomly selected patients who were not eligible were excluded and other patients were randomly selected to replace them. The eligibility criteria for breast cancer selected only records for women with newly referred localized breast cancer (*in situ* and invasive breast carcinoma) in 1994 and 1996. A record was considered to be assessable if surgical biopsy for breast cancer had been reported. We selected all the records for new colon cancer patients in 1994 and in 1996, but one centre in the intervention group did not have any patients with colon cancer, thus only six hospitals were analyzed for colon cancer. Eligibility criteria for colon cancer included newly referred women or men in 1994 and 1996, with any stage of cancer.

### Measurements

**Figure 1:**
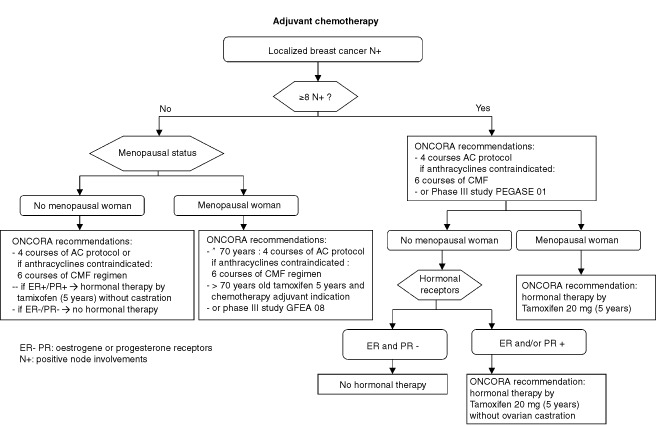
Example of decisional algorithms.

The main outcome was the number of overall treatment sequences judged to conform with the CPGs. The overall treatment sequence included the decisions for each type of procedure individually (surgery, radiotherapy, chemotherapy, hormonal therapy, initial examination, follow-up). Each of those procedures were assessed for conformity with the recommendations in the CPGs. Finally, the overall treatment sequence was judged to conform if all the assessable component procedures were followed. Only medical decisions covered by the CPGs were taken into consideration in the assessment of compliance. We decided to use the overall treatment sequences (i.e. a sequence of decisions along a management pathway) because it is possible that medical decisions by type of procedures were not independent of each other. For example, surgeons could present only patients records with localized breast cancer and node involvement to the medical oncologist for chemotherapy indication. However, results for each type of procedure individually were presented to understand why guidelines had not been followed. Figure 1

shows an example of these translated decisional algorithms of adjuvant chemotherapy indication for breast cancer patients with node involvement. Only the initial overall treatment sequence including surgery, radiotherapy, chemotherapy and hormonal therapy with initial examination and follow-up (when these were performed in the participating hospital) were taken into account. Hence, only one overall treatment sequence could be assessed for each patient. For colon cancer, data for both initial treatment and relapses were considered since CPGs were available for all phases of colon cancer management, hence, several overall treatment sequences could be assessed for colon cancer patients.

The second level of conformity was the number of medical decisions judged to be based on either the CPGs or published randomized trials i.e. ‘evidence-based’ by performing a systematic literature search using a documented strategy as developed by Sackett (1989). On the basis of the evidence unearthed in this way all types of procedure were individually classified by the blinded experts of the comprehensive cancer centre (one professor, six senior house officers, four residents and three students) as being ‘evidence-based’ if they complied with the CPGs' recommendations or were judged to be based on scientific evidence (scientific-level graded from I to II (Sackett, 1989)), or if they had been established in one or more RCTs or overviews of RCTs. When no published randomized trials were identified for at least one individual medical decision, the overall treatment sequences was classified as having ‘no convincing supporting scientific evidence’.

### Statistical analysis

Compliance was scored as 1 if the recommendations was followed and as 0 if not. Categorical data were analyzed using the Pearson χ^2^ or Fisher exacts test, as appropriate. Continuous data were analyzed with Student's *t*-test. The statistical significance level was set at *P*=0.05 in a two-sided test. Univariate analyzes were performed to assess if the patients' characteristics were correlated with compliance or non-compliance with the CPGs. A Mantel–Haenszel χ^2^ test (Mantel, 1963) stratified on centre was performed to assess concordance between the overall results and the individual hospital results. A Mantel–Haenszel χ^2^ test, stratified on the stage of the colon cancer was performed to evaluate the impact of the publication of the results for new chemotherapy regimens for the treatment of metastatic colon cancer (de Gramont *et al*, 1995, 1997) on medical practice.

## RESULTS (BREAST CANCER)

### Patients' characteristics

In the experimental group, 37 (10%) and 21 (6%) of the 319 and 367 records selected in 1994 and 1996, respectively, did not satisfy the selection criteria. In the control group 18 (8%) and 40 (19%) of the 212 and 210 records selected in 1994 and 1996, respectively, were not assessable for overall treatment sequence (Table 1

**Table 1:**
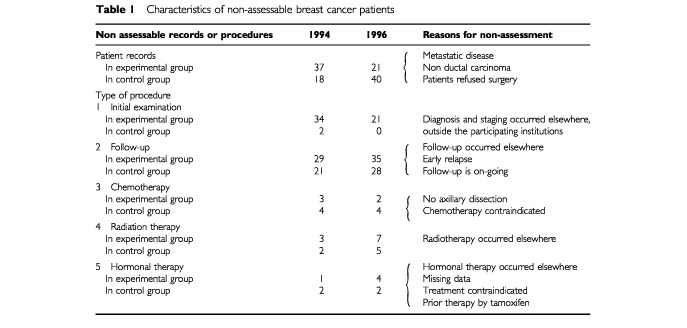
Characteristics of non-assessable breast cancer patients

).

For both groups of patients, some records were not assessable for certain type of procedures individually. Table 1 summarizes the reasons for non-evaluation of these procedures. For both groups of hospitals, the characteristics of the patients were similar for the 2 years (Table 2

**Table 2:**
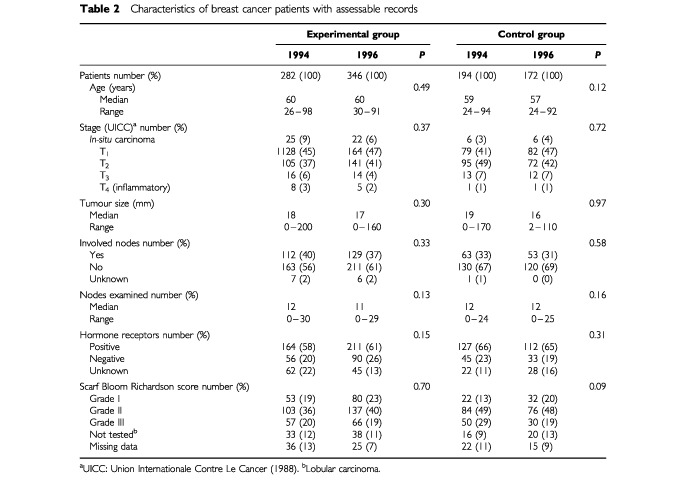
Characteristics of breast cancer patients with assessable records

).

### Compliance rate with CPGs

#### Experimental group

The observed compliance rate with CPGs for the 628 assessable overall treatment sequences was significantly higher in 1996 (36%; 125 out of 346) than that in 1994 (12%; 34 out of 282). In addition the number of individual medical decisions complying with the CPGs was also significantly higher in 1996 than in 1994 (Table 3

**Table 3:**
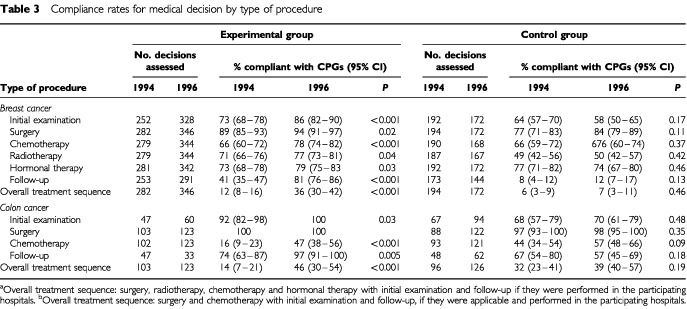
Compliance rates for medical decision by type of procedure

).

#### Control group

We detected no difference in the observed compliance rate with CPGs for the 366 assessable overall treatment sequences in 1996 (7%; 12 out of 172) and in 1994 (6%; 12 out of 194). The number of individual medical decisions complying with the CPGs was also similar for the 2 years (Table 3).

The compliance rate for surgery for patients aged 75 or over (79 out of 92 and 25 out of 46, in the experimental and control groups, respectively) was significantly lower than that for patients under 75 years of age (498 out of 536 and 269 out of 320, in the experimental and control groups, respectively) *P*<0.01).

The number of overall treatment sequences complying with the CPGs in each hospital in the experimental group was significantly higher in 1996 than in 1994 (results not shown). No change was noted for the hospitals in the control group (results not shown). The initial compliance rate with the CPGs i.e. in 1994, was significantly different between the control and the experimental groups of patients (6% *vs* 12%, *P*=0.03).

### ‘Evidence-based’ decisions and ‘no convincing scientific evidence’ decisions

For the experimental group, in 1994, 47% (95% CI, 41–53) (132 out of 282) and in 1996, 62% (95% CI, 54–64) (214 out of 346) (*P*<0.001) of initial treatments were conform with the CPGs or were judged to be based on scientific evidence. In the control group these results were 90 out of 194 in 1994 (46%; 95% CI, 39–53) and 80 out of 172 in 1996 (47%; 95% CI, 39–54), *P*=0.98. The medical decisions for 1994 and 1996, not covered by the CPGs but based on scientific evidence are summarized in Table 4

**Table 4:**
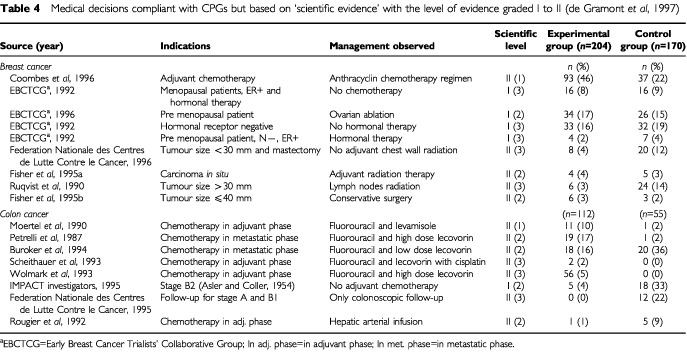
Medical decisions compliant with CPGs but based on ‘scientific evidence’ with the level of evidence graded I to II (de Gramont *et al*, 1997)

.

In 1994, 101 of the 282 medical decisions in the experimental group (36%; 95% CI, 30–42) were classified as having ‘no convincing scientific evidence’ compare with 72 out of 346 in 1996 (21%; 95% CI, 17–25), *P*<0.001. In the control group these results were 96 out of 194 in 1994 (49%; 95% CI, 42–56) and 87 out of 172 in 1996 (51%; 95% CI, 44–58), *P*=0.91.

These medical decisions concerned follow-up that was too intensive (132 patients in the experimental and 237 patients in the control group), or over-frequent measurement of tumour markers during follow-up. For chemotherapy, this was due to the prescription of regimens that were not validated in the adjuvant phase. For radiotherapy, this was due to the absence of adjuvant irradiation after conservative surgery for elderly patients, absence of sub-clavicular irradiation for an internal tumour site with nodal involvement, adjuvant irradiation of regional lymph nodes for an external small node negative tumour, and radiation therapy after axillary dissection. For hormonal therapy, this was due to absence of adjuvant treatment for menopausal patients with positive hormone receptors or adjuvant hormonal therapy for carcinoma *in situ*. For surgery, the reasons were no axillary dissection for elderly patients, or radical mastectomy for patients with a tumour smaller than 3 cm instead of conservative surgery and adjuvant irradiation; conservative surgery and adjuvant irradiation for patients with a tumour larger than 4 cm rather than radical mastectomy; no axillary dissection for management of plurifocal carcinoma *in situ* and conservative surgical treatment of retro-areolar breast tumours in the experimental group.

## RESULTS (COLON CANCER)

### Patients' characteristics

In the experimental group, 95 and 94 records for newly referred patients with colon cancer were eligible for inclusion in 1994 and 1996, respectively, corresponding to 103 and 96 overall treatment sequences. In the control group, 89 and 118 records for newly referred patients with colon cancer were eligible for inclusion in 1994 and 1996 respectively, corresponding to 96 and 126 overall treatment sequences. The CPGs contained no guidelines concerning initial examination and follow-up for metastatic disease, and, thus, only a few medical decisions were assessable for these aspects of practice. For the two groups (experimental and control) only 80 and 110 records, respectively, were assessable for follow-up, either because this occurred elsewhere, the patient had died, or early relapse at the time of the analysis. The patients from the hospitals in the experimental group treated in 1996 were significantly older than those seen in 1994 and more patients had more than one therapeutic sequence in 1996 than in 1994 (Table 5

**Table 5:**
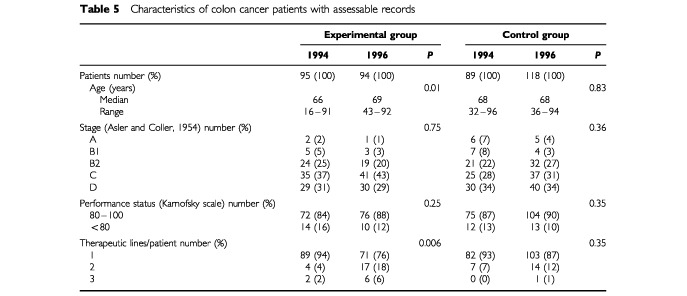
Characteristics of colon cancer patients with assessable records

). There were no differences in the patients from the control group hospitals.

### Compliance rate with CPGs

#### Experimental group

The observed compliance rate with CPGs for the assessable overall treatment sequences was significantly higher in 1996 (46%; 57 out of 123) than in 1994 (14%; 14 out of 103). In addition the number of individual medical decisions complying with the CPGs was also significantly higher in 1996 than that in 1994 except for surgical procedures (Table 3). The Mantel–Haenszel χ^2^ test stratified on stage (local *vs* metastatic) showed that the compliance rate for the overall treatment sequence was higher in 1996 than in 1994 for any stage of the disease (*P*<0.001) in this group.

#### Control group

We detected no difference in the observed compliance rate with CPGs for the assessable overall treatment sequences in 1996 (32%; 31 out of 96) and in 1994 (39%; 49 out of 126) *P*=0.19. The number of individual medical decisions complying with the CPGs was also similar for the 2 years (Table 3).

For both groups of patients in 1994 and in 1996, the results from univariate analyzes showed no statistically significant correlation between patients' characteristics (tumour markers, performance status, tumour stage, age, number of therapeutic lines per patients) and medical decisions.

The number of overall treatment sequences complying with the CPGs in each hospital in the experimental group was significantly higher in 1996 than in 1994 (results not shown). No change was noted for the hospitals in the control group (results not shown). The initial compliance rate with the CPGs i.e. in 1994, was significantly different between the control and the experimental groups of patients (14% *vs* 32%, *P*=0.01).

### ‘Evidence-based’ decisions and ‘no convincing scientific evidence’ decisions

For the experimental group, in 1994, 74% (95% CI, 65–82) (76 out of 103) and in 1996, 86% (95% CI, 80–92) (106 out of 123) of initial treatments were conformed with the CPGs or were judged to be based on scientific evidence, *P*<0.001. In the control group these results were 61 out of 96 in 1994 (64%; 95% CI, 54–74) and 75 out of 123 in 1996 (61%; 95% CI, 52–70), *P*=0.69. The medical decisions for 1994 and 1996, not covered by the CPGs but based on scientific evidence are summarized in Table 4.

In the experimental group, 26% (27 out of 103; 95% CI 17–34), and 17% (21 out of 123; 95% CI 10–23) (*P*=0.09) of the medical decisions in 1994 and 1996, respectively, were classified as ‘having no convincing scientific evidence’. In the control group 29% of the medical decisions (28 out of 96; 95% CI, 20–38) in 1994 and 24% (30 out of 126; 95% CI, 17–31) (*P*=0.40) in 1996 were judged as ‘having no convincing scientific evidence’. These decisions concerned adjuvant radiation therapy, and radiation therapy for liver metastases. The non-compliant medical decisions concerning chemotherapy procedures, were no adjuvant or palliative chemotherapy for elderly patients, prescription of adjuvant chemotherapy regimen for stage B1, absence of adjuvant chemotherapy for stage C and prescription of non-validated chemotherapy regimens instead of campthotecin.

## DISCUSSION AND CONCLUSIONS

In this controlled ‘before-after’ study of medical decisions we report an increase in the number of medical decisions compliant with CPGs in the experimental group compared with that in the matched control group. The baseline rates appeared to be very different, indicating that hospitals did not have the same baseline of conformed medical decisions for cancer management. However, it is the difference between the time periods for the two groups, which is important for the comparison rather than the comparison between the two groups. In the experimental group, for breast cancer the most frequent modification of practice concerned less extensive follow-up after treatment. More importantly, mastectomy rate for tumours smaller than 30 mm was zero. This approach of local management for early breast cancer had been shown to be efficient in randomized controlled trials since 1989 (Sarrazin *et al*, 1989). In 1994, 17% (9 out of 52) mastectomy were performed rather than conservative treatment in the experimental group *vs* 35% (24 out of 69) in the control group! Although there was a higher percentage increase of compliance with CPGs from 1994 to 1996 in the control hospitals than in the experimental hospitals for surgery (7% *vs* 5%), this was not statistically significant. The explanation is partly due to the rate of mastectomy for small tumours which decreased in the control group between 1994 and 1996 (24 out of 69, 35% *vs* 13 out of 49, 26%, respectively). For colon cancer, we observed a higher conformity with the CPGs in 1996 than in 1994 for all procedures except surgery. The most frequent modification was the choice of chemotherapy regimen and the less frequent modification was the use of chemotherapy. The median increase in compliance in the experimental group was 10% by type of procedures, which, although significant, remained modest, but the baseline compliance was superior to 70%. However, a conformity rate of 100% is probably impossible to obtain irrespective of the guidelines evaluated, essentially because of the patients' characteristics and preferences! For the overall treatment sequence, the baseline rate and the percentage in 1996 were less than 50% mainly because only one deviation in the procedure modify the compliance rate. This could be explained by the fact that follow-up care rarely deviates from conformity and that there were no major deviations for some patients and only minor deviations for many patients.

The results from this study suggest that the implementation of the CPGs in the network was effective, since modifications were observed compared with the practice in the control group. Interventions based on group dynamics and sensitive to the local practice context have been shown to be useful in facilitating the adoption of guidelines by physicians. Other methods, for example, consensus conferences, have been reported to have little or no effect on physicians in hospital practice (Kosecoff *et al*, 1987). The results observed in the network hospitals were similar to those previously reported for one comprehensive cancer centre (Ray-Coquard *et al*, 1997).

Although the conformity rates in 1994 in this multicentric study were lower than those reported for the single hospital, the rate increase was of a similar size. Moreover, this result was observed, despite the fact that the physicians in the other network hospitals did not elaborate the CPGs, although they had validated them. As in the previous study (Ray-Coquard *et al*, 1997), it is very encouraging to note for the experimental group that 62% of the medical decisions were evidence-based as defined by Ellis (Grimshaw and Russell, 1993) in 1996 which compares favourably with the 47% observed in 1994, although there was no modification in the control group (46% *vs* 47%). Although the practitioners in the network did not established that their decisions could be supported by evidence (this was retrospectively realized by the authors), the results of the study would suggest that introducing guidelines modifies medical practice, making it more conformed with the CPGs and also increased the conformity with evidence-based medicine. Establishing the infrastructure for practicing evidence-based medicine cost money (one million dollars each year for the organization of the ONCORA network) but practice guidelines and practicing medicine seems to be compatible.

It is most difficult to understand why practitioners in the network made evidence-based practice decisions that were not in accordance with the CPGs. For example, four cycles of AC (adriamycine plus cyclophosphamide) is the chemotherapy regimen recommended in adjuvant phase in the CPGs (Figure 1) but six cycles of FEC 100 (five Fluorouracile, epirubicin plus cyclophosphamide) (Coombes *et al*, 1996) was an ‘evidence-based chemotherapy regimen’ and for some young patients with very poor prognosis this attitude seems adapted without questioning the validity of the guidelines project. This kind of decision has always been very difficult to analyze retrospectively and interpretation leads to only hypotheses and not evidence.

In our study, the retrospective data collection may have offered some advantages over a prospective randomized study since, with a prospective data collection, there could have been ‘contamination’ in the control group, if the physicians had been aware of the study (Russell and Grimshaw, 1992; Ellis *et al*, 1995). In addition, the hospitals had volunteered and it would have been unrealistic to randomize the institutions and, then ask them to participate in the evaluation. Hence, we decided that a stratified analysis was necessary as seven centres were assessed. One limitation of this study would be a lack of comparability between the two regions. The control setting is about half the size of the experimental setting for most variables measured, although the number of new cancer patients managed every year was similar in each group and the population size was the most important predictor of decision quality and reproducibility (Glasgow *et al*, 1999).

We plan to assess if the diffusion of the results (feedback) presented here, will modify the medical practice of the physicians, since this has not always been reported to be effective (Lomas *et al*, 1991) and we also plan to assess the stability of this level of compliance over time, after the intensive implementation strategy has discontinued (Tierney *et al*, 1990). Future research should explore questions such as: what does making medical decisions more compliant with the CPGs mean? And how does this modify practice? For example, would changing the payment system modify prescription behavior (Rice, 1983)? A further problem is that there is no clear threshold of ‘acceptable’ compliance above which we could consider that the CPGs have been implemented well. It would be completely unrealistic to set this threshold at 100%, but we need to define a meaningful limit. These questions must be answered so that we can create more effective methods for involving physicians in quality assurance programmes.

The results in this study show that by adapting the external dissemination process, it is possible to reach a consensus for the CPGs in a target audience, and thus bring about a behaviour change (Delamothe, 1993). When applied to physicians in community hospitals, our educational strategy of opinion leaders performing ‘detailing based on the CPGs' produced a significant change in the behaviour.

## References

[bib1] AslerVBCollerFA1954 The prognostic significance of direct extension of cancer of the colon and rectum Ann Surg 139 846852 13159135 10.1097/00000658-195406000-00015 PMC1609522

[bib2] BurokerTRO'ConnellMJWieandHSKrookJEGerstnerJBMaillardJASchaeferPLLevittRKardinalCGGesmeJrDH1994 Randomized comparison of 2 schedules of fluorouracil and lecoverin in the treatment of advanced colorectal cancer J Clin Oncol 12 1420 7677801 10.1200/JCO.1994.12.1.14

[bib3] Centre Léon Bérard Réseau ONCORA1997 Thesaurus ONCORA en cancérologieArnette Blackwell: Paris

[bib4] CoombesRCBlissJMWilsJMorvanFEspieMAmadoriDet al1996 Adjuvant cyclophosphamide, methotrexate, and fluorouracil versus fluorouracil, epirubicin, and cyclophosphamide chemotherapy in premenopausal women with axillary node-positive operable breast cancer: Results of a randomized trial J Clin Oncol 14 3545 8558217 10.1200/JCO.1996.14.1.35

[bib5] DavisDAThomsonMAOxmanADHaynesRB1992 Evidence for the effectiveness of CME. A review of 50 randomized controlled trials JAMA 268 11111117 1501333

[bib6] de GramontABossetJFMilanCRougierPBouchéOEtiennePLet al1995 A prospectively randomized trial comparing 5-FU bolus with low dose folinic acid and 5FU bolus plus continuous infusion with high dose folinic acid for advanced colo-rectal cancer Proc Am Soc Clin Oncol 194 455(abstract)

[bib7] de GramontABossetJFMilanCRougierPBouchéOEtiennePLMorvanFLouvetCGuillotTFrancoisEBedenneL1997 Randomized trial comparing monthly low-dose leucoverin and fluorouracil bolus with bimonthly high-dose leucoverin and fluorouracil bolus plus continuous infusion for advanced colorectal cancer: a French intergroup study J Clin Oncol 2 808815 10.1200/JCO.1997.15.2.808 9053508

[bib8] DelamotheT1993 Wanted: guidelines that doctors will follow Br Med J 307 218 8369678 10.1136/bmj.307.6898.218 PMC1678177

[bib9] Early Breast Cancer Trialists' Collaborative Group (EBCTCG)1992 Systematic treatment of early breast cancer by hormonal, cytotoxic or immune therapy Lancet 339 115 1345950

[bib10] Early breast Cancer Trialists' Collaborative Group (EBCTCG)1996 Ovarian ablation in early breast cancer: overview of the randomized trials Lancet 348 11891196 8898035

[bib11] EddyDM1990 Guidelines for policy statements: the explicit approach JAMA 263 22392243 2319689 10.1001/jama.263.16.2239

[bib12] EddyDM1991 The individual vs. Society: is there a conflict? JAMA 265 14461450 1999888 10.1001/jama.265.11.1446

[bib13] EllisJMulliganIRoweJSackettDL1995 In patient general medicine is evidence based Lancet 346 407410 7623571

[bib14] FarrowDCHuntWCSametJM1992 Geographic variation in the treatment of localized breast cancer N Engl J Med 326 10971101 1552910 10.1056/NEJM199204233261701

[bib15] Federation Nationale des Centres de Lutte Contre le Cancer1995 Standards, Options, Recommandations (SOR) vol 2 Cancers digestifspp 88151Paris: Arnette Blackwell SA

[bib16] Federation Nationale des Centres de Lutte Contre le Cancer1996 Standards, Options, Recommandations (SOR) vol 3 Cancers du sein non metastatiquespp 107127Paris: Arnette Blackwell SA

[bib17] FergusonJH1995 The NIH consensus development program Jt Comm J Qual Improv 21 332336 7581735 10.1016/s1070-3241(16)30157-2

[bib18] FinkAKosecoffJChassinMBrookRH1984 Consensus methods: characteristics and guidelines for use Am J Public Health 74 979983 6380323 10.2105/ajph.74.9.979 PMC1651783

[bib19] FisherERConstantinoJFisherBPakelarASRedmondCMamounasE1995a Pathologic findings from the National Surgical Adjuvant Breast Project (NSABP) Protocol-B17. Intraductal carcinoma. The National Surgical Adjuvant Breast and Bowel Project Collaborating Investigators Cancer 75 13101319 7882281 10.1002/1097-0142(19950315)75:6<1310::aid-cncr2820750613>3.0.co;2-g

[bib20] FisherBAndersonSRedmondCWolmarkNWickermanDLCroninWM1995b Reanalyses and results after 12 years of follow-up in randomized clinical trial comparing total mastectomy with lumpectomy with or without irradiation in the treatment of breast cancer N Engl J Med 333 14561461 7477145 10.1056/NEJM199511303332203

[bib21] GlasgowREShowstackJAKatzPPCorveraCUWarrenRSMulvihillSJ1999 The relationship between hospital volume and outcomes of hepatic resection for hepatocellular carcinoma Arch Surg 134 3035 9927127 10.1001/archsurg.134.1.30

[bib22] GrilliRLomasJ1994 Evaluating the message: The relationship between compliance rate and the subject of a practice guideline Med Care 32 202213 8145598 10.1097/00005650-199403000-00002

[bib23] GrimshawJMRussellIT1993 Effect of clinical guidelines on medical practice: a systematic review of rigorous evaluations Lancet 342 13171322 7901634 10.1016/0140-6736(93)92244-n

[bib24] International Multicentre Pooled Analysis of Colon Cancer Trials (IMPACT) investigators1995 Efficacy of adjuvant fluorouracil and folinic acid in colon cancer Lancet 345 939944 7715291

[bib25] KosecoffJKanouseDERogersWHMcCloskeyLWinslowCMBrookRH1987 Effects of the National Institutes of Health Consensus Development Program on physician practice JAMA 258 27082713 3499522

[bib26] LomasJEnkinMAndersonGMHannahWJVaydaESingerJ1991 Opinion leaders vs. audit and feedback to implement practice guidelines JAMA 265 22022207 2013952

[bib27] MantelN1963 Chi-square tests with one degree of freedom; extension of the Mantel-Haenszel procedure J Am Stat Assoc 58 690700

[bib28] McDonaldCJHuiSLSmithDMTierneyWMCohenSJWeinbergerMMcCabeGP1984 Reminders to physicians from an introspective computer medical record. A two-year randomized trial Ann Intern Med 100 130138 6691639 10.7326/0003-4819-100-1-130

[bib29] MoertelCGFlemingTRMacdonaldJSHallerDGLaurieJAGoodmanPJUngerleiderJSEmersonWATormeyDCGlickJH1990 Levamizol and fluorouracil for adjuvant therapy of resected colon carcinoma N Engl J Med 322 352358 2300087 10.1056/NEJM199002083220602

[bib30] PetrelliNHerreraLRustumYBurkePCreavenPStulsJEmrichLJMittelmanA1987 A prospective randomized trial of 5-fluorouracil versus 5-fluorouracil and high-dose leucoverin versus 5-fluorouracil and methotrexate in previously untreated patients with advanced colorectal carcinoma J Clin Oncol 5 15591565 2443619 10.1200/JCO.1987.5.10.1559

[bib31] Ray-CoquardIPhilipTLehmannMFerversBFarsiFChauvinF1997 Impact of a clinical guidelines program for breast and colon cancer in a French cancer center JAMA 278 15911595 9370505

[bib32] RiceTH1983 The impact of changing Medicare reimbursement rates on physician-induced demand Med Care 21 803815 6350746 10.1097/00005650-198308000-00004

[bib33] RougierPLaplancheAHuguierMHayJMOllivierJMEscatJSalmonRJulienMRoullet AudyJCGallotD1992 Hepatic arterial infusion of floxuridine in patients with liver metastases from colorectal carcinoma: long-term results of a prospective randomized trial J Clin Oncol 10 11121118 1296590 10.1200/JCO.1992.10.7.1112

[bib34] RutqvistLECedermarkBGlasUJohanssonHRotsteinSSkoogLSomellATheveTWilkingNAskergrenJ1990 Randomized trial of adjuvant tamoxifen combined with postoperative radiation therapy or adjuvant chemotherapy in postmenopausal breast cancer Cancer 66 8996 2191764 10.1002/1097-0142(19900701)66:1<89::aid-cncr2820660117>3.0.co;2-g

[bib35] RussellITGrimshawJM1992 The effectiveness of referral guidelines: a review of the methods and findings of published evaluationsInHospital ReferralRolland M, Coulter A (eds)pp 179211Oxford: Oxford University Press

[bib36] SackettDL1989 Rules of evidence and clinical recommendations on the use of antithrombotic agents Chest 95 2S4S 2914516

[bib37] SarrazinDLeMArriagadaRContessoGFontaineFSpielmannMRochardFLe ChevalierTLacourJ1989 Ten year results of randomized trial comparing a conservative treatment to mastectomy in early breast cancer Radiother Oncol 14 177184 2652199 10.1016/0167-8140(89)90165-5

[bib38] ScheithauerWRosenHKornekGVSebestaCDepishD1993 Randomized comparison of combination chemotherapy plus supportive care with supportive care alone in patients with metastatic colorectal cancer Br Med J 306 752755 7683942 10.1136/bmj.306.6880.752 PMC1677246

[bib39] SirenPBLaffelGl1996 Quality management in Managed careInThe Managed Health Care HandbookKongstvedt PR (ed)pp 403426Gaithersburg, USA: Aspen

[bib40] TierneyWMMillerMEMcDonaldCJ1990 The effect on test ordering of informing physicians of the charges for outpatient diagnostic tests N Engl J Med 322 14991504 2186274 10.1056/NEJM199005243222105

[bib41] Union Internationale Contre Le Cancer (UICC) TNM1988InClassification des tumeurs malignes4th edn,Paris (eds)pp 99105Springer-Verlag

[bib42] WolmarkNRocketteHFisherBWickerhamDLRedmondCFisherERJonesJMamounasEPOreLPetrelliNJ1993 The benefit of lecoverin-modulated fluorouracil as postoperative adjuvant therapy for primary colon cancer: results from National Surgical Adjuvant Breast and Bowel Project protocol C-03 J Clin Oncol 11 18791887 8410113 10.1200/JCO.1993.11.10.1879

